# Amyotrophic Lateral Sclerosis and Its Management during the COVID-19 Pandemic: A Qualitative Study with Thematic Analysis of Patients and Caregivers Who Participated in Self-Help Groups

**DOI:** 10.3390/bs13100822

**Published:** 2023-10-05

**Authors:** Lorenza Palazzo, Laura Pizzolato, Matteo Rigo, Giuseppina Bondì

**Affiliations:** Department of Philosophy, Sociology, Pedagogy and Applied Psychology (FISPPA), University of Padova, 35139 Padova, Italy; laura.pizzolato.1@studenti.unipd.it (L.P.); matteo.rigo.5@studenti.unipd.it (M.R.); giusybond@gmail.com (G.B.)

**Keywords:** amyotrophic lateral sclerosis (ALS), COVID-19 pandemic, self-help groups, caregivers, telemedicine, qualitative study

## Abstract

This study employs a qualitative methodology to explore the effects of the pandemic on the lives of ALS patients and their caregivers. It aims to understand whether and how online self-help groups have assisted families dealing with amyotrophic lateral sclerosis (ALS) patients. ALS is a neurodegenerative disease with both physical and psychosocial implications. Consequently, it significantly affects the lives of patients’ caregivers. In 2020, the COVID-19 pandemic exacerbated this situation. The results show that the pandemic has had a negative impact on the well-being of ALS caregivers and patients. Furthermore, bereavement and death were dealt with in different ways by the families involved. The pandemic aggravated the health of ALS patients and increased the workload of their caregivers; however, online psychological support was appreciated for its role in providing emotional help and diminishing social isolation.

## 1. Introduction

Amyotrophic lateral sclerosis (ALS) is a progressive neurodegenerative disease of motor neurons [1]. The illness clinically manifests as atrophy, muscle weakness, spasticity, and fasciculations, and it progresses rapidly in multiple regions of the body without periods of remission [2]. It is also often accompanied by cognitive dysfunction (in 50% of cases) and/or behavioral dysfunction (in 70% of cases) [3]. The progression of the disease leads to the weakening of the body’s voluntary muscles, which reaches a state of total immobility and respiratory failure [4].

Living with an incurable disease such as ALS can induce negative thoughts and concerns about physical, psychosocial, and existential issues [5]. The emotional distress triggered by the illness is primarily attributed to the loss of independence and the gradual decline in functions and abilities associated with it [6,7]. Patients frequently grapple with emotions like fear, embarrassment, shame, distress, anger, guilt, and a profound sense of hopelessness. Notably, some patients express a desire for assisted suicide [8,9].

Living with ALS results in a continuous experience of loss and uncertainty, affecting not only patients but also their family members, especially their caregivers [10,11]. In most cases, a family member assumes the role of caregiver, often designated by other family members who entrust them with the care responsibilities; in over 70% of cases, this caregiver is the spouse [12,13]. The impact on their emotional and physical health, personal and social quality of life, and financial status is referred to in the literature as the ‘burden of care’. This burden is associated with the caregiver’s workload, behavior, and functional impairment [14].

The burden of care on ALS caregivers becomes notably significant, particularly with the progression of the disease [15,16]. Factors that exert the most influence on the perceived burden include the fear of an uncertain future, increased responsibilities, social life disruptions, physical limitations, and constraints on time and personal freedom [17].

In the face of heightened burden, family caregivers frequently find themselves compelled to cut down on the time and attention they allocate to their personal needs, leisure pursuits, and broader family and social connections [18,19]. The rising demand for caregiving often necessitates leaving employment or scaling back working hours, leading to reduced income and limited career prospects [20]. Consequently, caregivers may experience emotions such as anger, anxiety, stress, fear, frustration, and uncertainty linked to their caregiving role [5,15]. Moreover, as ALS progresses, patients encounter a growing loss of autonomy in their daily activities, rendering them increasingly reliant on their caregivers [21].

The COVID-19 pandemic intensified the distress experienced by ALS patients and their caregivers [22]. Within the general population, the psychological repercussions of this health crisis manifested as heightened anxiety across various aspects of human interaction due to the perceived loss of control over one’s living environment [23,24]. Social isolation and loneliness emerged as prominent adverse outcomes of the pandemic for ALS patients and their caregivers, becoming primary concerns during lockdown. Additionally, there was a fear of being forgotten and abandoned by healthcare professionals [25,26]. Additionally, a more rapid decline in functional status was observed during periods of restrictive measures [27]. The most notable changes were associated with disturbances in cognitive functions and emotional states, deterioration in well-being and quality of life, disrupted sleep patterns, and unhealthy eating behaviors, particularly among women [28].

During the COVID-19 pandemic, numerous ALS centers transitioned to telemedicine to ensure continuous care for patients and their caregivers [7]. In light of this shift, certain studies have underscored the challenges faced in facilitating physical activities at home and coordinating online exercise groups or telerehabilitation interventions [29,30]. However, other studies have reported advantages, including the swift identification of potentially critical situations and timely prescription of aids [31], remote psychological and speech counseling [32], disease progression monitoring [33], and the reduction of both time and financial costs associated with traveling to ALS centers [34].

The aim of the current study was to delve into the ramifications of the COVID-19 pandemic on ALS patients and their caregivers. This exploration centered on discerning the coping mechanisms employed during the health emergency and subsequent lockdowns, particularly concerning disease management. Additionally, the study aimed to ascertain the effectiveness of online psychological support for both patients and caregivers during the lockdown period. To achieve these goals, a qualitative research design was implemented, employing thematic analysis on semi-structured interviews to delve into the intricacies of experiences related to the illness and the accompanying emotions.

## 2. Materials and Methods

### 2.1. Research Design

**Figure 1:**
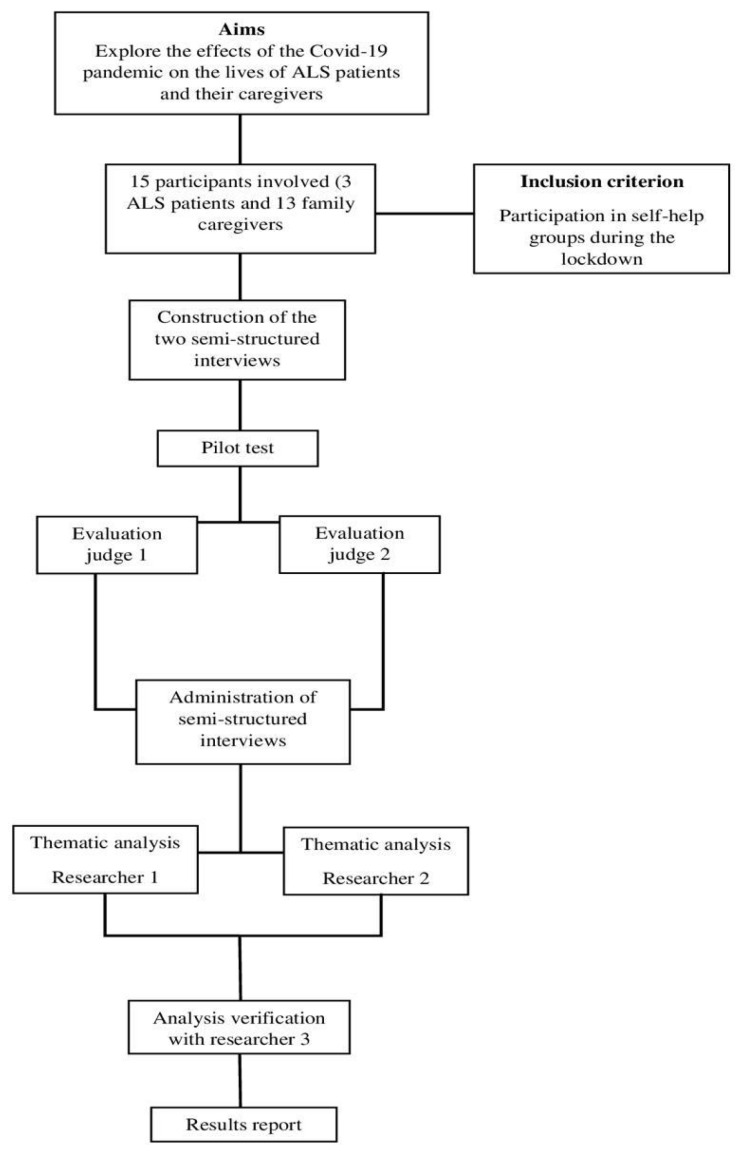
Block diagram of the study.

A qualitative research approach was employed to delve into the experiences and emotions linked to the illness (Figure 1), following the guidelines outlined in the Consolidated Criteria for Reporting Qualitative Research (COREQ) Checklist [35,36].

Data were gathered via two meticulously designed semi-structured interviews, one tailored for the patients and another for the caregivers. These interviews were specifically crafted and conducted by two researchers who had received specialized training. Importantly, there was no pre-existing acquaintance between the researchers and the participants, ensuring an unbiased and objective data collection process. Semi-structured interviews were deliberately selected to delve deeply into the participants’ experiences and viewpoints on the subject matter. This approach facilitated meticulous data collection, providing rich and detailed information vital for achieving a profound comprehension of the phenomenon under investigation. To ensure the effectiveness of the interview questions, a pilot test was executed. Initially, these questions were administered to three individuals who were not included in the research sample. This preliminary evaluation guaranteed the clarity and appropriateness of the questions before their implementation in the actual interviews.

The pilot test served the purpose of identifying and resolving potential issues or ambiguities in the interview questions. It also aimed to gauge the estimated duration of the interview, evaluate the clarity and effectiveness of the questions, and assess the participants’ comprehensibility and responsiveness. Furthermore, the pilot test helped in determining the suitability of the interview approach for obtaining the desired information and ensuring participants’ comfort in responding to the questions.

Following this, the same set of questions underwent evaluation by two impartial judges proficient in the field of ALS. Their expertise was utilized to gauge the relevance of these questions.

For instance, questions posed to the patients included inquiries such as, “How has the COVID-19 pandemic and the subsequent lockdown impacted your management of the disease? In what manner?” and “Do you believe you can offer guidance to families undergoing a situation similar to yours during these challenging times?”.

Certain questions tailored for the caregivers encompassed queries such as, “In what aspects of illness management did you encounter the most challenges? Conversely, which aspects did you find manageable, and what contributed to this ease?” and “Did any alterations occur in family dynamics throughout the lockdown period?”. Additionally, caregivers were asked, “Do you perceive the support received from the association, particularly through the self-help groups, played a role in alleviating your sense of isolation during the lockdown? If so, in what manner?”.

The semi-structured interviews were carried out individually amid the initial lockdown phase, transpiring in Italy during the spring of 2020. These interviews were facilitated remotely, employing either telephone conversations or video calls, and were meticulously recorded to facilitate subsequent transcription.

Upon the interview’s completion, participants were granted a brief interval of approximately 10 min. During this time, they were prompted to confirm or refute their earlier responses. This step aimed to ascertain the accuracy and completeness of their answers, ensuring they authentically reflected the participants’ experiences and opinions.

Subsequently, a thematic analysis of the textual data was performed utilizing a bottom-up approach, as outlined by previous literature [37]. This method enabled us to dynamically identify emerging themes within the data. The analysis provided a meticulous account, emphasizing the significance of these themes in relation to our research inquiries [38].

Thematic analysis was selected due to its established reputation as a robust method for exploring and pinpointing key themes within qualitative data. This approach offers exceptional flexibility, particularly when dealing with research questions demanding interpretative and exploratory efforts, as evident in this study. It proved instrumental in comprehending participants’ experiences and generating pertinent insights to address the research queries effectively.

Following the structured guidelines delineated by Braun and Clarke [39], the interviews underwent meticulous transcription and comprehensive review, enhancing our familiarity with their contents.

Following this, a comprehensive codebook was meticulously curated, incorporating meaningful excerpts extracted from each interview. Initial codes were systematically generated, and hierarchical relationships were delineated. These codes were subsequently organized into themes and macro-themes directly pertinent to the research question. In the subsequent phase, a rigorous revision process was undertaken to assess the relevance of all identified themes. Subsequently, these themes were meticulously defined and labeled, culminating in the creation of a thematic map. This map served as a comprehensive guide, elucidating the core essence of each theme identified in the analysis.

Finally, a report was compiled, incorporating an analytical narrative [37]. The coding process was supported using Atlas.ti software [40]. Two researchers conducted the analysis simultaneously, comparing and subsequently merging their findings. Subsequently, they met with a third member to verify the consistency and robustness of their analyses, resolve any discrepancies, and ultimately reach an agreement on thematic generation.

This study adhered to the code of conduct outlined by the American Psychological Association and followed the principles of the Declaration of Helsinki. All participants received a comprehensive explanation of the study’s objectives and methodology. They provided signed informed consent forms, granting authorization for their participation and approving all aspects of the interview protocol. Participants were asked to record the interviews for transcription and content analysis purposes, with the assurance that the content would be kept confidential. The study received approval from the University of Padua’s Experimental Ethics Committee (reference: C3DD8C5FCE1C26C7E80954B4EC34DC16).

### 2.2. Participants

The study encompassed a cohort of 15 participants, comprising 3 ALS patients (all male) and 12 family caregivers (10 females, constituting 83.33%, and 2 males, representing 16.67%). The patients exhibited an average age of 54 years (SD = 9.02), while the caregivers had an average age of 70 years (SD = 11.47). Convenience sampling, a method falling within the realm of non-probability sampling, was employed to enlist participants affiliated with an Italian patient association. Notably, all participants hailed from Northern Italy. The inclusion criterion mandated participation in the self-help groups organized during the lockdown, facilitated by two psychologists affiliated with the patient association. Importantly, engagement in the interview process was voluntary and entirely independent of any services offered by the association. The socio-demographic details of both caregivers and patients are presented in Table 1 and Table 2, respectively. To ensure the confidentiality and anonymity of the participants, fictitious names have been employed throughout the article.

## 3. Results

### 3.1. The ALS Patients

After a meticulous analysis of the data collected from interviews with individuals affected by ALS (amyotrophic lateral sclerosis), our examination revealed three distinct thematic areas that emerged as significant aspects of their experiences during the research period (Table 3). These thematic areas were identified as follows: (Section 3.1.1) The Impact of the Pandemic, (Section 3.2.2) ALS Management, and (Section 3.2.3) Online Support.

These three thematic areas proved pivotal in comprehending the experiences and viewpoints of ALS patients within the context of the research.

#### 3.1.1. The Impact of the Pandemic

Initially, the pandemic instigated profound fear of the virus and frustration due to the time lost, as evidenced by the statements: “Fear, primarily, arose due to the limited knowledge about COVID-19” and “Dismay—struck me when I witnessed the coffins being transported on military trucks”. Simultaneously, the lockdown restrictions provided a sense of protection from the virus, allowing the patients to perceive themselves as more aligned with everyone else. The latter phenomenon stemmed from the patients’ ability to conceal their differences during the lockdown, sparing them from the embarrassment associated with their diminished abilities. Lorenzo elucidated this sentiment, stating, “The necessity to stay at home, to avoid public appearances, to limit my interactions solely to my immediate family—that was acceptable. It was uncomplicated”.

Secondly, the pandemic ushered in a series of alterations in everyday and family life. Daily and work routines necessitated reorganization, personal spaces had to be shared more, and time had to be allocated differently. Concurrently, family bonds were fortified. Tommaso expressed these sentiments, stating, “Having my daughters at home all the time hadn’t occurred in a few years. It was pleasant to adjust my spaces to accommodate the presence of two additional individuals”.

#### 3.1.2. Dealing with ALS

Concerning the disease and its progression, participants revealed feelings of discomfort, frustration, and shame stemming from their inability to perform fundamental actions. Additionally, they grappled with emotions of anger, fear, and uncertainty regarding the future. These emotions are vividly portrayed in the following statements: “I don’t know how long I will endure” and “I perceive the future as obscure, vague, and challenging to decipher”. The challenges posed by the illness primarily revolved around diminished mobility and a compromised sense of personal autonomy. In this context, Lorenzo articulated: “I’m never alone when I venture outside; there’s always someone accompanying me. I rely on a machine that transports me to work, to the individuals I visit, or any destination I need to reach”.

Patients employed diverse strategies to cope with the limitations imposed by the disease: fostering a positive and constructive mindset to mitigate the distress stemming from an uncertain and grim future, devising practical solutions to handle the practical and physical challenges induced by ALS, and, ultimately, coming to terms with and embracing the inevitability of death. Tommaso’s words epitomize the latter aspect:

“I am convinced that death is the final moment of one’s life. I understand that life, when it concludes, simply ceases to exist […]. I discussed it calmly with my wife and children, conveying my tranquility to them”.

#### 3.1.3. Online Support

Despite the challenges posed by the pandemic, participants regarded the online self-help groups as a crucial resource providing emotional support and fostering a sense of closeness and camaraderie. This sentiment is reflected in their responses: “It enabled me to converse with individuals grappling with the same illness as mine, albeit in unique ways” and “I felt that someone extended a hand”.

For certain patients, the online self-help groups facilitated a sense of independence and autonomy. As an illustration, Lorenzo expressed: “When the groups revert to in-person meetings, I might encounter more challenges […] because participation will entail going out, confronting the world, navigating physical spaces, and requesting someone to accompany me—a complete system that isn’t necessary at the moment. Currently, I am entirely self-sufficient”.

### 3.2. The Family Carers

A thorough thematic analysis of the data from caregiver interviews revealed three crucial areas that encapsulated their experiences and challenges (Table 4). These areas can be delineated as follows: (Section 3.2.1) The Impact of the Pandemic, (Section 3.2.2) The Burden of Care, and (Section 3.2.3) Online Support.

These three thematic areas emerged as pivotal in our comprehension of the caregiver’s perspective, particularly within the context of the challenges presented by the pandemic and the evolving landscape of caregiving.

#### 3.2.1. The Impact of the Pandemic

The caregivers expressed a significant psychological burden characterized by confusion, sadness, frustration, anger due to the limited time they could devote to their loved ones, feelings of loneliness, and distress. Floriana described her experience as follows: “I felt destabilized because everything that used to work—my routine—was no longer applicable. [I also felt] sadness, undoubtedly, but more so due to what I had to do—the effort, the constant adjustments demanded of my habits”.

In certain instances, the sadness and sense of helplessness escalated into anxiety and depression, as highlighted by several participants. Two examples include: “A prevailing sense of discouragement that persisted for an extended period” and “There were phases when I felt overwhelmingly anxious about everything”. Notably, only two participants reported feeling a sense of protection and being treated on par with the rest of the population. The predominant emotion remained the fear of contracting the virus and potentially infecting loved ones, as elucidated by Erica: “I fear the illness. Simple as that. I fear the illness. I don’t want people in the house. I don’t allow them in”.

Consequently, face-to-face interactions diminished, resulting in profound loneliness and a substantial lack of emotional connection with family members. Reflecting on this aspect, Giulia expressed, “Perhaps the emotion I experienced the most was despair—anguish. Yes, because I was distant […]. Being deprived of [personal] contact is a chilling and inhumane experience”.

Furthermore, the measures implemented to curb the virus’s spread necessitated additional technical and welfare support for families. Erica recounted her experience: “I found myself alone because the individual who used to visit me briefly in the mornings got frightened and ceased coming”.

Nevertheless, the lockdown ushered in novel relational dynamics within families. It enabled caregivers to allocate more time to themselves, fostering increased sharing of personal spaces and time. However, in some instances, this situation gave rise to tensions and unease, exacerbated by the absence of external distractions and the inability to host guests at home. Giorgia, for instance, shared her perspective on these aspects: “Releasing that tension becomes considerably more challenging when you’re confined to your home and unable to go out to interact with others and engage in exchanges”.

#### 3.2.2. The Burden of Care

The progression of ALS poses a myriad of emotional challenges for caregivers. Experiencing helplessness, fearing inadequacy, and enduring the constant fatigue that accompanies the daily management of the disease constitute a significant emotional burden.

Certainly, one of the most distressing aspects for caregivers is the breakdown in communication caused by dysarthria. In these moments, caregivers can sense that their loved ones have something important to express, yet the words remain unintelligible. Beatrice vividly conveyed this frustration, stating, “There were times when we felt utterly helpless because she clearly had something to say—something on her mind—but we couldn’t comprehend each other. That was incredibly frustrating for me”.

Confronting an incurable disease invariably brings forth the looming presence of death, a subject often left unspoken within families. Giorgia disclosed, “Honestly, we never discussed any plans. I actually learned about Giulio’s true intentions when he conveyed them to the palliative therapist. So, you could say there are certain taboos. Death is undoubtedly one of them, and it’s not a topic we broach”. When discussions about death did take place within the family, they initially stirred profound pain. However, for many caregivers, engaging in this uncomfortable conversation became a necessary step toward acceptance.

Two distinct perspectives on death surfaced among caregivers. Some perceived it as a transition, opting to find solace in the idea that death is a natural part of life’s progression. Giorgia articulated, “Finding peace with death, viewing it as a transition rather than the ultimate end. Seeing death as a component of life”. In contrast, others regarded death as a complete cessation, a form of obliteration. Lucia articulated this viewpoint, stating, “Spiritually, I believe it’s the end […]. The person lingers in our thoughts and memories, but I consider it the end—unfortunately”.

An ALS diagnosis can evoke profound fear and confusion, accompanied by uncertainty and apprehension about the future. Marco pondered on this uncertainty, stating, “The future is incredibly uncertain. We are aware that there will be a final event, but we don’t know when. We witness our loved ones deteriorate—in my case, progressively—but in a gradual and unpredictable manner. I am uncertain about how long they will live and how I should prepare for the [ultimate] moment and its aftermath”.

Amidst the challenges presented by both the disease and the pandemic, caregivers sought practical information, aid, and emotional support. They redirected their focus to the present and embraced a positive perspective on the future as coping mechanisms. Some also found comfort in spiritual and religious beliefs, as Sara expressed, “All the suffering he endured cannot be meaningless; it just can’t… I firmly believe it’s not an end in itself. It was for something greater—an afterlife, a place where he will receive what he did not receive here on Earth”.

This comprehensive approach enabled caregivers to navigate the challenges of caregiving and find significance in their experiences of suffering, loss, and eventual death.

#### 3.2.3. Online Support

The online self-help groups were viewed as beneficial and positive, as they helped alleviate the feeling of isolation and maintained the continuity of the weekly meetings. Specifically, the online format addressed the needs voiced by the caregivers, encompassing emotional and informational support, the opportunity to share and compare experiences, and a sense of normality and familiarity with others. These elements contributed to the empowerment of the participants. Matteo expressed this sentiment, stating, “The self-help group has been an invaluable source of guidance and support throughout the years, and especially so during this time. In one of the virtual meetings, I shared my feelings of isolation, and I emerged stronger knowing that I was truly understood”.

Giulia described her experience with the group in the following way: “Being able to see and talk to people who were experiencing this same ordeal was incredibly significant for me. I felt like I was adrift at sea, but then I found a life jacket and could say, ‘Okay, I can stay afloat.’”

Meeting other caregivers who had experience with the same disease was valuable for solving practical daily issues and acquiring information about the progression of the illness. The latter aspect helped to alleviate the sense of uncertainty regarding what the disease would entail in the future. Floriana shared her experience: “When I found myself in a complex situation and had to make a decision I was unsure about, I talked to a peer, which was helpful. Unfortunately, we still know very little about ALS, so we often struggle in the dark before understanding what can be done, what challenges the patient will face, and where to seek help. Basically, it’s all still too vague. The association’s self-help group has been incredibly helpful, even on a practical level”.

For certain participants, the group provided a space where they could shed their caregiver role and freely share their emotional experiences with genuine empathy. Chiara articulated this sentiment:

“Occasionally, I strip away my nurse attire and simply become a daughter who has lost a mother, entitled to feel anger, sadness, and happiness. If I wish to talk, I do; if I prefer silence, I embrace it”.

## 4. Discussion

The COVID-19 pandemic posed unique challenges for ALS care and research, leading to diagnostic delays and challenges in monitoring disease progression and providing multidisciplinary care [41]. These issues were primarily caused by the restrictions implemented to curb the virus’s spread [42]. To address these challenges, diverse digital tools, including telemedicine, were employed to ensure the continuation of effective care [33,43].

Consistent with existing literature [44,45,46], this study has affirmed a substantial psychological impact on caregivers. This impact was notably heightened during the pandemic, leading to feelings of sadness and helplessness. These emotions were triggered by the unpredictability of the situation, the challenges in adapting to new caregiving demands, and a diminished quality of life [27,47,48]. Concerning the patients, the interviews predominantly unveiled fear and frustration due to the time lost while being confined at home. They also experienced a sense of unease and uncertainty about the future, emotions commonly linked with the disease and its progression [49,50,51]. Nonetheless, the lockdown also triggered positive feelings, including a sense of protection from contagion and a perception of equality, as everyone faced reduced social interactions and fewer external demands [9].

Moreover, the findings indicate the emergence of new relational dynamics, marked by collaboration among family members, increased emotional closeness, and enhanced practical support for patients. However, there were also moments of tension and nervousness due to the stress induced by the disease and the restricted social interactions. As previously suggested by studies, the family plays a pivotal role as one of the most significant psychosocial factors that enables patients to cope with the challenges posed by the disease [52,53].

The study delved into the role of online support offered by self-help groups. In these virtual environments, patients and caregivers could exchange experiences, information, and solutions [54]. Patients found these groups beneficial, providing psycho-emotional and informational support, fostering a sense of closeness and sharing, and promoting empowerment and autonomy [33]. Simultaneously, these groups addressed caregivers’ needs to temporarily set aside their roles and express their emotional experiences freely and empathetically. This support network helped mitigate feelings of isolation and uncertainty concerning the future progression of the illness.

Numerous studies have highlighted that healthcare professionals frequently overlook caregivers’ need for support [55,56]. Additionally, information regarding prognosis and disease progression is often inconsistent or inadequate [19], a situation exacerbated by the absence of a shared language regarding death in contemporary society [57,58].

According to the results, patients endeavored to navigate the challenges posed by the disease and the pandemic by employing task- and solution-oriented coping strategies, embracing attitudes centered on the present, and maintaining positive thinking. These findings resonate with recent studies that underscore patients’ inclination to employ coping strategies rooted in the creation of new meanings and the positive reinterpretation of challenging situations. Such strategies serve to alleviate anxiety about the future and the inevitability of death [7,9,59,60]. The progression of the disease results in a growing loss of control for ALS patients [8]. Consequently, concentrating on the present emerges as a valuable strategy for preserving autonomy concerning the illness and one’s life [61]. Caregiving, being exceptionally demanding and overwhelming, compels caregivers to fortify their coping mechanisms and cultivate resilience [45]. Participants demonstrated a readiness to cherish the invaluable relationships with their loved ones in the present moment, seeking assistance, emotional support, and information.

Finally, this study delved into the experiences of ALS patients and their caregivers concerning the topic of death. Some families managed to confront and accept this reality after enduring profound suffering. Conversely, for other families, death remained an unspoken taboo, inducing intense anxiety at the moment of diagnosis [62,63,64]. Two distinct perspectives on death surfaced within the sample. The first regarded it as the ultimate end, while the second perceived it as a transitional moment leading to the afterlife. This latter interpretation was intertwined with spiritual and religious coping strategies, acting as protective factors that enabled individuals to find fresh meaning amid suffering and loss [8,65,66]. Several other studies conducted during the pandemic have discovered the effectiveness of religion and spirituality in mitigating the negative effects of the health emergency [67], aiding coping with chronic illnesses [68], providing comfort, and promoting well-being [69,70].

The main limitations of this study were the small sample size of patients (only three individuals) and its gender composition (solely males), rendering the results non-generalizable.

## 5. Conclusions

The current study revealed a decline in the health status of ALS patients during the COVID-19 pandemic, coupled with a heightened caregiving burden among caregivers. Concurrently, the study observed new positive relational dynamics within families, marked by enhanced closeness, sharing, and a sense of belonging to the wider community. Regarding online psychological support, the self-help groups were positively evaluated by both patients and caregivers for providing emotional and informational support, effectively combating feelings of social isolation.

This study’s strengths lie in its nuanced approach to complex issues surrounding the impact of the COVID-19 pandemic and lockdown on both ALS patients and their caregivers. The analysis conducted during this unique period provides a valuable opportunity for healthcare professionals, researchers, and health authorities to closely observe the impact of lockdown measures on the progression of ALS and caregiving management by caregivers and family members. This observation is vital for anticipating potential consequences of future health restrictions and adapting strategies to meet the evolving needs of patients. This includes the development of more effective online interventions tailored specifically to meet the diverse needs of users.

Indeed, the diversity within the caregiver sample, both in terms of gender and family situations, poses a challenge to the study’s generalizability. Consequently, future research endeavors would greatly benefit from a larger and more homogenous sample of ALS patients and caregivers. To yield more nuanced insights, employing a mixed-methods approach incorporating standardized instruments for quantitative data collection could prove immensely valuable. This methodological refinement would enhance the study’s precision and offer a more comprehensive understanding of the intricate dynamics at play within this context. Moreover, future studies could include comparisons between the periods before and after the pandemic or between individuals who received support and those who did not. Despite these limitations, this study offers valuable insights into the impact of the pandemic and lockdown on ALS patients and caregivers. These findings can be instrumental in shaping future healthcare policies and guiding clinical practices and research.

## Figures and Tables

**Table 1:** Caregivers’ demographic characteristics.

Fictitious Name	Gender	Age	City of Residence	Degree	Employment	Family Relation with ALS Patient	Deceased Family Member
Floriana	F	47	Milan	Graduated	Employed	Spouse	
Beatrice	F	53	Milan	High school diploma	Housewife	Mother	x
Giorgia	F	52	Milan	Graduated	Self-employed	Spouse	
Matteo	M	68	Pisa	Graduated	Company director	Spouse	x
Giulia	F	46	Milan	Graduated	Teacher	Mother	x
Roberta	F	41	Milan	Graduated	Manager	Father	x
Marco	M	76	Milan	Graduated	Manager	Spouse	
Angelica	F	73	Milan	High school diploma	Retired	Spouse	
Chiara	F	47	Milan	Graduated	Nurse	Mother	x
Lucia	F	60	Empoli	High school diploma	Employed	Father	
Erica	F	66	Borgo S. Lorenzo (Florence)	High school diploma	Retired	Spouse	
Sara	F	58	Empoli	High school diploma	Employed	Spouse	x

**Table 2:** Patients’ demographic characteristics.

Fictitious Name	Gender	Age	City of Residence	Degree	Employment	Stage of ALS	Time of Diagnosis
Massimo	M	55	Milan	Graduated	Lawyer	Advanced stage (Non-Invasive Ventilation h24)	October 2018
Lorenzo	M	45	Milan	Ph.D.	Researcher and University Professor	Initial stage (slow progression)	July 2019
Tommaso	M	63	Florence	High school diploma	Company director	Advanced stage (Palliative care activated)	June 2020

**Table 3:** ALS patients’ results.

ALS Patients	Exemplars
**Impact of the pandemic**	◦ ** Emotional Impact ** The pandemic triggered intense emotions among patients, including fear of the unknown and anger at time lost. ◦ ** Safety During Lockdown ** Lockdown restrictions made patients feel more protected from the virus and allowed them to hide their differences. ◦ ** Changes in Daily Life ** The pandemic led to significant changes in daily life and family dynamics, requiring a reorganization of routines and different sharing of spaces and time. ◦ ** Strengthening Family Bonds ** Restrictions strengthened family bonds, leading family members to spend more time together.
**ALS management**	◦ ** Initial Reactions to the Pandemic ** Initial fear, anger, and uncertainty due to the pandemic. ◦ ** Feeling of Security during Lockdown ** During the lockdown, patients felt more protected from the virus. ◦ ** Dealing with the Illness ** Patients experienced discomfort and frustration due to a loss of autonomy.They developed strategies to cope with difficulties, including positive thinking, practical solutions, and acceptance of death.
**Online support**	◦ ** Online Self-Help Groups as a Valuable Resource ** Participants viewed online self-help groups as vital resources during the pandemic.These groups offered emotional support and a sense of connection, providing a platform for sharing experiences. Some participants appreciated the opportunity to interact with others who had the same illness, albeit in a different way. ◦ ** Enhancing Independence ** Online self-help groups allowed some patients to maintain a sense of independence and autonomy.

**Table 4:** Family carers’ results.

Family Carers	Exemplars
**Impact of the pandemic**	◦ **Psychological Impact on Caregivers** Caregivers experienced confusion, sadness, frustration, anger, loneliness, and distress due to the pandemic. ◦ **Fear of Infection** The primary fear among caregivers was the fear of being infected by the virus and transmitting it to their loved ones, leading to reduced in-person contact and profound loneliness. ◦ **Changed Family Dynamics** - The lockdown led to new relational dynamics within families, with more time devoted to self and increased sharing of personal spaces and time. - However, in some cases, this caused tensions and nervousness due to the lack of external distractions and the inability to receive guests at home. ◦ **Need for Technical and Welfare Support** Measures to contain the virus required increased technical and welfare support for families.In some instances, this support decreased due to concerns related to the virus.
**Burden of care**	◦ **Emotional Challenges for Caregivers** Caregivers face emotional challenges such as helplessness, fear of inadequacy, and constant fatigue due to managing ALS. ◦ **Communication Difficulties** Dysarthria in ALS patients leads to breakdowns in communication, causing frustration and a sense of helplessness among caregivers. ◦ **Taboo of Discussing Death** Death remains a taboo topic within families, and conversations about it often cause pain. Some caregivers view death as a natural transition, while others see it as annihilation. ◦ **Uncertainty About the Future** An ALS diagnosis brings uncertainty and concern about the future as caregivers witness the slow deterioration of their loved ones. ◦ **Coping Strategies** Caregivers seek practical information, assistance, and emotional support.They adopt a positive outlook on the future and may find solace in spiritual and religious beliefs.
**Online support**	◦ **Reduces isolation.** ◦ **Offers emotional support and practical information.** ◦ **Provides a sense of normalcy and familiarity.** ◦ **Empowers participants to tackle ALS-related challenges.** ◦ **Helps manage uncertainty about the future.** ◦ **Serves as an outlet for emotional expression**

## Data Availability

The datasets used during the current study are available from the corresponding author on reasonable request.
